# INSR gene variation is associated with decreased insulin sensitivity in Iraqi women with PCOs

**Published:** 2014-07

**Authors:** Manal T. Mutib, Farqad B. Hamdan, Anam R. Al-Salihi

**Affiliations:** 1 *The High Institute of Infertility Diagnosis and Assisted Reproductive Technology, Al-Nahrain University, Baghdad, Iraq.*; 2 *Department of Physiology, College of Medicine, Al-Nahrain University, Baghdad, Iraq*

**Keywords:** *PCOS*, *Insulin receptor gene*, *GTT*, *lipid proteins*, *Iraqi female*

## Abstract

**Background:** Polycystic ovarian syndrome (PCOS) is a complex, heterogeneous disorder of uncertain etiology with strong genetic background. Insulin resistance is present in the majority of PCOS cases with linkage and association between single nucleotide polymorphisms of insulin receptor (INSR) gene and PCOS.

**Objective:** To examine whether the exon 17 of INSR gene contributes to genetic susceptibility to PCOS in Iraqi women and its effects on glucose tolerance test and lipid profile.

**Materials and Methods: **Sixty-five healthy Iraqi women and eighty-four infertile women with PCOS, divided into two subgroups depending on the BMI were studied. Restriction fragment length polymorphism (RFLP-PCR) analysis was performed to determine the genotypes for the His 1058 C/T polymorphism at the tyrosine kinase domain in the INSR gene. Clinical, anthropometric and biochemical parameters were also estimated.

**Results: **The C/T polymorphism at His 1058 in exon 17 of INSR was associated with PCOS (obese and non-obese). CC genotype frequency was higher in PCOS patients whereas TT genotype was higher in control women. Those with CC genotype had higher BMI, GTT and lipid profile than those with TT genotype.

**Conclusion:** An association of C/T polymorphism at His1058 of INSR with PCOS in Iraqi women was observed. Its association with indices of insulin resistance and dyslipidemia were also noticed.

## Introduction

Polycystic ovary syndrome (PCOS) is one of the most common female endocrine disorders of reproductive age with principal features of anovulation i.e., irregular menstruation, amenorrhea, infertility; masculinizing features i.e., acne and hirsutism; insulin resistance, obesity, Type 2 diabetes, and high cholesterol levels (1). The etiology of PCOS is still unknown, there is increasing evidence to support a major genetic basis, since the syndrome is strongly familial (2). It is clear, however, that more than one gene (and probably several) contributes to the heterogeneous phenotype (3). 

The metabolic abnormalities is now well recognized as a growing public health problem in PCOS, there will be increased risk of central obesity, hypertension, glucose intolerance, hyperinsulinemia, low serum level of high-density lipoprotein- cholesterol and high serum level of triglycerides. People with metabolic abnormalities are at increased risk for type 2 diabetes mellitus (T2DM) and cardiovascular disease (CVD). More evidences have shown that insulin resistance is an underlying pathophysiologic defect in PCOS (4).

Insulin resistance may occur secondary to resistance at the insulin receptor, decreased hepatic clearance of insulin, and/or increased pancreatic sensitivity (5). Insulin receptor is a heterotetrameric glycoprotein, it is a member of tyrosine kinase receptor family, consists of two α and β dimmers that are linked by disulfide bonds and encoded by the *INSR *located at the chromosome 19p13.2, composed of 22 exons and spans greater than 120 kilo base pairs (kbp) (6). The region of exons 17-21 encodes the tyrosine kinase domain of the receptor, which is necessary for insulin signal transduction. Mutation in these exons has been shown to cause severe insulin resistance and hyperinsulinemia (7). Binding of an insulin molecule activates the kinase activity of the receptor, and auto phosphorylation of specific tyrosine residues occurs (8). 

Several kinds of polymorphisms have been identified within the coding and noncoding regions of *INSR *in patients with PCOS. Of these polymorphisms, most were silent single-nucleotide polymorphisms (SNPs) and there was a higher frequency of SNP in exon 17 of *INSR* (9). It was stated that the number and affinity of *INSR* is not altered in PCOS but its tyrosine phosphorylation status and subsequent signaling is affected, suggesting the defect may lie in the β-chain (10). Among the SNPs in exon 17 of *INSR *detected to date the C/T SNP at His1058 in the tyrosine kinase domain containing the ATP binding site of *INSR *has been shown to be associated with the development of PCOS most possibly by the resultant effects on the auto phosphorylation of the *INSR* function in some women with PCOS (6, 9, 10). The intention of our study was to examine whether the exon 17 of *INSR* gene contributes to genetic susceptibility to the PCOS in Iraqi women and its effects on glucose tolerance test and lipid profile.

## Materials and methods

A case-control study conducted in the High Institute of Infertility Diagnosis and Assisted Reproductive Technologies and Al-Nahrain Forensic DNA Unit, Baghdad, Iraq for the period extended from February 2012 to February 2013. The study was approved by the Local Medical Ethical Committee of the College of Medicine, Al-Nahrain University, and written consent was obtained from patients or their surrogates to participate in the study. One hundred and forty nine women were studied comprised of 65 healthy women served as the control group and 84 women whom diagnosed as having PCOS. The latter group had history of oligomenorrhea and evidence of hyperandrogenism (on clinical examination or by documented elevated testosterone levels). Women with any other cause of oligomenorrhea and hyperandrogenism were excluded. 

We enrolled only women who had PCO on ultrasonography to ensure that the phenotype was definitely PCOS. Clinical and biochemical characteristics of women with PCOS and controls are given in Table 1. According to BMI, each groups were subdivided in two subgroups (obese ≥30 kg/m^2^ and non-obese <30 kg/m^2^). Basal blood samples were obtained from the studied subjects to measure plasma FSH, LH, E2, testosterone hormones, fasting blood sugar (FBS), blood glucose level after half, one and two hours. The cholesterol, triglyceride, low-density lipoprotein (LDL), very low-density lipoprotein (VLDL) and high-density lipoprotein (HDL) were measured.

**Genetic analysis**

Blood samples for molecular genetic studies were collected in tubes containing EDTA as an anticoagulant. We extracted the genomic DNA from the blood of patients with PCOS and the control women by using gene extraction kit supplied by Geneaid Company (Thailand). The restriction fragment length polymorphism analysis was performed to determine genotypes for the His 1085 C/T polymorphism at the tyrosine kinase domain in the *INSR *gene. 

Exon 17 was amplified by polymerase chain reaction (PCR), using forward primer: CCAAGGATGCTGTGTAG ATAAG, reverse primer: TCAGGAAAGCCAG CCCATGTC. A total volume of 50 µl containing genomic DNA 100 ng was used as template in the reaction mixture, 10 mol of each primer and 25 µl of Green Master Mix (Promega, USA). Cycling parameters were denaturated at 94^o^C for 5 minutes, 35 cycles with 94^o^C for 45 seconds, 55^o^C for 40 seconds, 72^o^C for 60 seconds, and 72^o^C for 10 min. PCR products (317-bp) digested with *PmlI* (Thermo Scientific, USA) for 16 hours at 37^o^C. 

**Figure 1:**
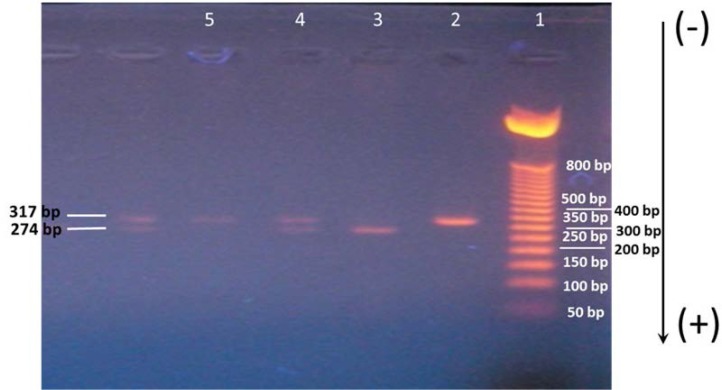
Restriction fragment length polymorphism analysis of the C/T polymorphism of exon 17 in the INSR gene. Agarose gel (2%) electrophoresis after *PmlI* digestion of the PCR

Digested DNA fragments were electrophoresed on a 2% agarose gel containing ethidium bromide and visualized by UV trans-illuminator spectroline (USA). Hence, a single 317-bp band indicates homozygosity for the TT genotype. The presence of two fragments, 274-bp and 43-bp bands, indicates homozygosity for the CC genotype. The presence of three fragments, 317-, 274-, and 43-bp bands, indicates heterozygosity for the CT genotype (Figure 1).

**Statistical analysis**

Statistical analysis was performed by using statistical package of Science (SPPS); Version 17.0 and, Microsoft Excel Worksheet 2010. Numerical data analysis was done by using unpaired sample t-test for tables with mean and standards deviation of mean to compare PCOS and control subjects. Chi-square was used to exam the significance of gene and alleles distribution in the two major groups and the subgroups. ANOVA test was used to test the anthropometric parameters and its relation to genes. The differences between values were considered statistically significant at the level of p<0.05.

Lane 1: 50 bp DNA ladder

Lane 2: PCR product (317-bp)

Lane 3: CC genotype (274-bp)

Lane 4: CT genotype (317-bp, 274-bp and 43-bp)

Lane 5: TT genotype (317-bp)

## Results

The demographic data of the PCOS patients and control women were illustrated in table I. As shown in table II, the genotyping distributions of exon 17 of INSR genes; the CC, CT and TT genotypes were significantly different in PCOS patients from that of the control women (p=0.002). Similarly, the genotypes of the obese PCOS patients were also different from that of the obese control women (p=0.034). No significant difference in the distribution of these genotypes was observed between non-obese PCOS patients and non-obese control women (p=0.056). On the contrary, no significant difference was found between the obese and non-obese PCOS patients (p=0.325). In the same manner, no difference was noticed in the genotypic distribution of the obese from non-obese control women (p=0.682).

Concerning the allelic frequency of exon 17 of INSR gene, PCOS patients presented high frequency of CC and low frequency of TT genotypes (p=0.001; p=0.013, respectively) but not with CT genotype of the exon 17 of INSR gene when compared to the control women (Table III). In addition, obese PCOS patients demonstrated higher frequency of C alleles (p=0.012) but not the CT and TT genotypes of exon 17 of INSR gene in comparison to obese control women. On the contrary, the frequency of these genotypes was not different between obese PCOS and non-obese PCOS (Table IV). Table IV also illustrates that the frequency of CC genotype was higher (p=0.041) in the non-obese PCOS vs. non-obese control women. On the other hand, the frequency of CT and TT genotypes were not different between the two groups. No difference was noticed in the frequency of these genotypes between obese and non-obese control subjects. According to the frequency of CC, CT and TT alleles, the BMI was 31.3±5.01, 27.98±4.75, and 27.74±3.7, respectively, and the waist/hip ratio was 0.84±0.06, 0.8±0.09, and 0.81±0.07, respectively. 

These values were significantly different in the PCOS patients (p=0.0082, p=0.0236, respectively). On the contrary, no significant was found in the control women. The FBS level was 101.71±11.89 mg/dl, 88.82±15.89 mg/dl, and 83.57±13.5 mg/dl and the blood sugar level measured after half hour was 164.4±30.41 mg/dl, 134.46±37.4 mg/dl, and 125.43±32.59 mg/dl, after one hour was 162.89±25.99 mg/dl, 125.36±26.62 mg/dl, and 113.29±32.48 mg/dl, and after two hours was 129.34±22.7 mg/dl, 100.93±19.38 mg/dl, and 92.57±17.23 mg/dl). These values show significant difference in PCOS patients according to the frequency of their genotype (p=0.0003, p=0.0008, p=0.0000, p=0.0000, respectively). In the control group, FBS level was 94±12.42mg/dl, 85.08±11.11mg/dl, and 76.6±12.41mg/dl, respectively), moreover, the blood sugar level measured after half hour was 141.91±19.5 mg/dl, 131.91±10.07 mg/dl, and 105±11.56 mg/dl, respectively. 

These values were different (p=0.0253, p=0.0049; respectively) according to the genotypic distribution. No difference was observed in the blood sugar level measured after one and two hours. Considering the lipid profile, PCOS patients showed significant difference in the triglyceride level (132.08±30.76 mg/dl, 114.89±30.88 mg/dl, 108.29±37.66 mg/dl respectively) and VLDL level (26.41±6.14 mg/dl, 22.69±6.39 mg/dl, and 22.00±7.15 mg/dl with respect to INSR genotypes (CC, CT and TT) (p=0.0473, p=0.0419; respectively). On the reverse, no difference was noticed in the cholesterol, LDL and HDL levels. In the same manner, the control women demonstrated significant difference in the triglyceride level (124.18±19.67 mg/dl, 106.96±21.15 mg/dl, and 96.13±23.71 mg/dl, VLDL level (24.84±3.93 mg/dl, 21.39±4.23 mg/dl, and 19.23±4.74 mg/dl, and HDL level 39.24±1.09 mg/dl, 41.35±2.57 mg/dl, and 42.13±2.07 mg/dl with respect to the CC, CT, and TT genotypes (p=0.0060, p=0.0061, p=0.0016, respectively). Meanwhile, no difference was observed in the cholesterol and LDL levels.

**Table I:** Comparison of demographic parameters between PCOS and control groups (using unpaired T test)

**Parameters**	**PCOS patients (N = 84)**	**Control women (N = 65)**	**p-value**
Age (yrs)	29.02 ± 4.50	30.31 ± 3.71	0.0584
BMI (kg/m^2^)	29.9 ± 5.13	28.10 ± 4.51	0.0270
Waist/ Hip Ratio	0.82 ± 0.07	0.80 ± 0.06	0.0382
Waist/thigh ratio	1.42 ± 0.18	1.33 ± 0.11	0.0002
FSH (IU/ml)	5.35 ± 1.55	7.22 ± 2.32	0.0000
LH (IU/ml)	6.24 ± 3.00	3.97 ± 1.55	0.0000
LH/FSH	1.20 ± 0.52	0.56 ± 0.15	0.0000
E_2_ (pg/ml)	60.27 ± 19.68	50.28 ± 18.90	0.0181
Testosterone (ng/ml)	0.79 ± 0.44	0.26 ± 0.15	0.0000
E_2_/Testosterone	103.22 ± 58.34	230.62 ± 132.57	0.0000
FBS (mg/dl)	94.81 ± 15.30	87.07 ± 12.78	0.0132
BS (after 1/2 hr.) (mg/dl)	148.53 ± 36.84	133.08 ± 18.77	0.0092
BS (after 1 hr) (mg/dl)	142.79 ± 33.16	124.63 ± 17.39	0.0007
BS (after 2 hr) (mg/dl)	112.71 ± 24.32	99.12 ± 11.97	0.0005
Cholesterol (mg/dl)	163.70 ± 30.72	140.92 ± 17.87	0.0000
Triglyceride (mg/dl)	124.68 ± 33.51	111.25 ± 23.09	0.0109
VLDL (mg/dl)	24.85 ± 6.76	22.25 ± 4.62	0.0140
LDL (mg/dl)	98.90 ± 29.36	77.98 ± 17.36	0.0000
HDL (mg/dl)	39.11 ± 2.49	40.73 ± 2.30	0.0004

Numbers are presented as mean±SD.

PCOS: polycystic ovary syndrome

FSH: follicular stimulating hormone

LH: luteinizing hormone

E_2_: estradiol

FBS: fasting blood sugar

BS: blood sugar

VLDL: very low density lipoprotein

LDL: low density lipoprotein

HDL: high density lipoprotein.

**Table II:** Distribution of C/T Alleles of exon 17 of INSR in classified PCOS patients and control women (using Chi square test)

**Groups**	**INSR genotypes**			**p-value**
	**CC**	**CT**	**TT**	
PCOS patients	44 (52.38)	32 (38.1)	8 (9.52)	0.002
Control women	17 (26.15)	32 (49.23)	16 (24.62)	
Obese PCOS patients	21 (61.77)	11 (32.35)	2 (5.88)	0.034
Obese Control women	6 (27.27)	12 (54.55)	4 (18.18)	
Non-obese PCOS patients	23 (46)	21 (42)	6 (12)	0.056
Non-obese Control women	11 (25.58)	20 (46.51)	12 (27.91)	
obese PCOS patients	21 (61.77)	11 (32.35)	2 (5.88)	0.325
Non-obese PCOS patients	23 (46)	21 (42)	6 (12)	
Obese Control women	6 (27.27)	12 (54.55)	4 (18.18)	0.682
Non-obese Control women	11 (25.58)	20 (46.51)	12 (27.91)	

Number are presented as n (%).

INSR: insulin receptor

PCOS: polycystic ovary syndrome

**Table III:** Allele frequencies of C/T polymorphism of exon 17 of INSR in PCOS patients and control group (using Chi square test)

**INSR genotypes**	**PCOS patients (N = 84)**	**Control women (N = 65)**	**p-value**
CC	44 (52.38)	17 (26.15)	0.001
CT	32 (38.1)	32 (49.23)	0.173
TT	8 (9.52)	16 (24.62)	0.013

Numbers are presented as n (%).

PCOS: polycystic ovary syndrome.

**Table IV:** Allele frequencies of C/T polymorphism of exon 17 of INSR in PCOS patients versus control group (obese and non-obese) by BMI (Using Chi square test)

**Groups**	**INSR genotypes**					
	**CC**	**p-value**	**CT**	**p-value**	**TT**	**p-value**
Obese PCOS patients	21 (61.77)	0.012	11 (32.35)	0.099	2 (5.88)	0.146
Obese control women	6 (27.27)		12 (54.55)		4 (18.18)	
Non-obese PCOS patients	23 (46)	0.041	21 (42)	0.662	6 (12)	0.053
Non-obese control women	11 (25.58)		20 (46.51)		12 (27.91)	
Obese PCOS patients	21 (61.77)	0.156	11 (32.35)	0.371	2 (5.88)	0.348
Non-obese PCOS patients	23 (46)		21 (42)		6 (12)	
Obese control women	6 (27.27)	0.883	12 (54.55)	0.540	4 (18.18)	0.389
Non-obese control women	11 (25.58)		20 (46.51)		12 (27.91)	

Numbers are presented as n (%).

PCOS: polycystic ovary syndrome.

## Discussion

Concerning molecular genetic studies, PCOS is one of the most extensively studied endocrinopathies in women, and attention has been given to insulin resistance, since many patients with PCOS have increased susceptibility of T2DM and show symptoms such as glucose intolerance and insulin resistance (11). Therefore, genes related to insulin action with special focus on the *INSR* gene have been suggested to be candidate for PCOS. 

The *INSR* receptor gene comprises 22 exons spanning 120 Kb on chromosome 19. Numerous single nucleotide polymorphisms for *INSR* gene were identified, the most important of them at exon 17 (12). Mutation at this region which encodes the tyrosine kinase domain of the insulin receptor was demonstrated to initiate severe insulin resistance and hyperinsulinemia and considerably associated with PCOS (13, 14). In this study, we analyzed the association between the His 1058 C/T polymorphism in the exon 17 of the *INSR *gene Iraqi PCOS women where an association between a single nucleotide polymorphism at exon 17 of the *INSR *gene and PCOS was observed. The frequencies of CC, CT and TT genotypes were significantly different between PCOS patients and control women (Table II).

Interestingly, TT genotype was higher in the control women than in the PCOS patient while CC genotype was higher in PCOS patients (Table III); a finding that also reported by Lee *et al* (15). Moreover, obese and non-obese PCOS patients frequently exhibited CC genotype while the frequency of TT genotype was clearly higher in obese and non-obese control women (15). This indicates that, regardless of the presence or absence of obesity; the frequency of the CC genotype was increased in PCOS patients compared to control women although the frequency of these genotypes did not differ significantly between both obese and non-obese PCOS and control.

Two previous studies indicate that the His 1085 C/T polymorphism at the tyrosine kinase domain of the *INSR *gene was demonstrated in lean white PCOS patients (9, 14). This study proved clear effect of C/T polymorphism in the exon 17 of the *INSR *gene on anthropometric and biochemical parameters (PCOS patients with CC genotype) had higher glucose level compared to those with other genotypes; in other words to have insulin resistance. In cases with mutations in exons 17-21, the region that encodes the tyrosine kinase domain of the insulin receptor, have been shown to cause severe insulin resistance and hyperinsulinemia (16). Excessive phosphorylation of serine residues of the *INSR* and downstream signaling molecules have been identified as a molecular cause of insulin resistance. This molecular defect reduces the tyrosine kinase activity of the *INSR*, thereby decreasing the signal transduction pathway (16, 17).

Studies of *INSR* function in some women with PCOS have detected changes in autophosphorylation that may have been secondary to polymorphisms in the tyrosine kinase domain which selectively affects metabolic but not mitogenic pathways in classic insulin target tissues and in the ovary (18). BMI and waist to hip ratio were higher in PCOS patients and control women with CC genotype. This is due to the clear association between CC genotype and abnormal glucose tolerance test, i.e., blood glucose level was elevated and the resultant compensatory hyperinsulinemia. 

Insulin induces obesity by acting on brain causing hungry. Recent evidence indicates that the brain processes information from adiposity signals such as insulin, which circulate in proportion to body fat mass, and integrates this input with signals from nutrients such as fatty acids (19, 20). In response, the brain sends signals to control feeding behavior and substrate metabolism in ways that promote homeostasis of both energy stores and fuel metabolism, and acts on liver to manufacture fat by converting extra calories to fat, and on the fat cells in muscle belly to be filled with fat through decreasing mitochondrial function (21, 22).

As noticed earlier, PCOS patients with CC genotype have insulin resistance and interestingly at the same time they show impaired lipid profile (high cholesterol, triglyceride, VLDL, LDL and low HDL). Insulin is a critical regulator of virtually all aspects of adipocyte biology, and adipocytes are one of the most highly insulin-responsive cell types. Insulin promotes adipocyte triglyceride stores by a number of mechanisms, including fostering the differentiation of preadipocytes to adipocytes and, in mature adipocytes, stimulating glucose transport and triglyceride synthesis (lipogenesis), as well as inhibiting lipolysis. It also increases the uptake of fatty acids derived from circulating lipoproteins by stimulating LPL activity in adipose tissue (23). In case of insulin resistance as discussed above there will be stimulation of lipolysis, altered expression of low protein lipase and hepatic lipase, increasing hepatic gluconeogenesis and inhibiting glucose uptake and oxidation in skeletal muscle (24, 25).

## Conclusion

In conclusion, a single nucleotide polymorphism in the tyrosine kinase domain of the INSR gene was associated with insulin resistance and atherogenic lipoprotein that have a role in the pathogenesis of PCOS in Iraqi women. 

## Footenote

***This article extracted from Ph.D. thesis. (Manal T. Mutib)***
